# Multireference Correlated Oscillator Strengths from Adiabatic Connection Approaches Based on Extended Random Phase Approximation

**DOI:** 10.1021/acs.jctc.4c00103

**Published:** 2024-04-26

**Authors:** Daria Drwal, Katarzyna Pernal, Ewa Pastorczak

**Affiliations:** Institute of Physics, Lodz University of Technology, ul. Wolczanska 217/221, 93-005 Lodz, Poland

## Abstract

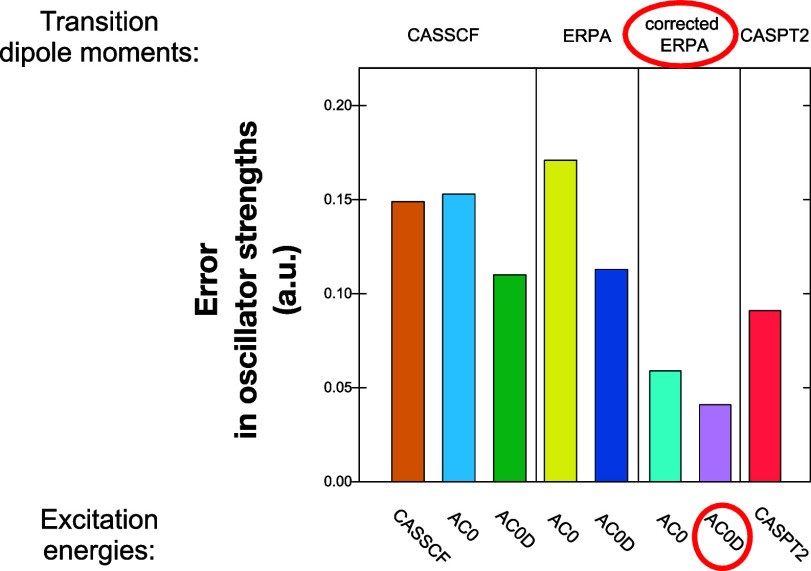

We show that accurate oscillator strengths can be obtained from adiabatic connection (AC) approaches based on the extended random phase approximation (ERPA) combined with multireference (complete active space, CAS) wave functions. The oscillator strengths calculated using the perturbation-corrected ERPA transition density matrices, proposed in this work, and the excitation energies calculated with recently introduced AC correlation energy methods, AC0 and AC0D, compete with accuracy in the perturbational CASPT2 approach and require less computational effort. AC0 and AC0D methods scale more favorably with the number of active orbitals than multiconfigurational perturbation approaches like CASPT2 and NEVPT2 thanks to their dependence on reduced density matrices up to the order of 2. Importantly, the newly developed approach for computing correlated transition dipole moments does not entail any additional costs, as all intermediate quantities become available when AC0 energies are being computed. We also test the performance of the recently proposed AC method corrected for the negative-transition contributions to the correlation energy, AC0D, for triplet excitation energies. Similarly, as for the singlet excitations, the correction improves the performance of the AC0 method, particularly for the low-lying excited states.

## 1 Introduction

One of the essential goals of quantum chemistry methods is the simulation of the absorption and emission spectra of molecules. This requires accurate computation of the electronic excitation energies and the oscillator strengths of electronic transitions.

In particular, obtaining the correct excitation energies requires a balanced description of both the static and dynamic correlation. The effects related to the static correlation can be described using a sufficiently flexible multiconfigurational wave function such as the complete active space (CAS) wave function, including the description of the dynamic correlation in a robust way can be challenging and computationally costly. To that end, multiple approaches combining wave function and density functional theories were developed.^1−9^ However, the most popular methods of including the dynamic correlation involve perturbation corrections like CASPT2 and NEVPT2.^10−14^

Despite their overall good performance, these methods are not without their problems. CASPT2, in particular, may suffer from the lack of size consistency^15^ and the intruder state problem which requires empirical shifting. The cost of CASPT2 and NEVPT2 computations scales unfavorably with the active space size, which limits their use to cases with around 20 active electrons. Notable attempts to capture the dynamic correlation for systems with multiple strongly correlated electrons include perturbation approaches developed for density matrix renormalization group,^16−20^ reduced configuration interaction,^21,22^ and full configuration interaction quantum Monte Carlo^23^ as well as driven similarity renormalization group^24^ references. Other approaches involve using coupled cluster (see e.g., ref (25)) or MRCI^26^ framework. For a comprehensive review of the multireference methods for excited states, see e.g., ref (27).

When it comes to the oscillator strengths, usually the perturbation correction is only used for correcting the excitation energy, although it was shown that using perturbatively modified CAS wave function for computing the transition dipole moments (TDMs) often improves their accuracy.^28^

Recently, we have proposed an alternative approach to computing the dynamic correlation for multireference wave functions, one rooted in the adiabatic connection (AC) fluctuation–dissipation theory.^29−32^ The AC approach combined with Extended Random Phase Approximation (ERPA)^33,34^ results in a set of computational methods that, unlike CASPT2 or NEVPT2, only require the construction of reduced density matrices of order as low as two. The performance of the AC-ERPA method and its more computationally efficient approximate variant, AC0, is comparable to CASPT2 and NEVPT2 methods for the ground states^30^ and low-lying triplet excitations^35,36^ but tends to overestimate the singlet excitation energies.^8^ We have recently rewritten the AC correlation energy formula in the framework of linear response theory and shown that it lacks the terms arising from the negative-energy transitions in the linear response function.^32^ We proposed a way of accounting for the missing terms in the AC0 approximation, and we presented the resulting method’s, AC0D, enhanced performance for the singlet excitations.

In the current paper, we investigate how the AC-based approaches, including the newly introduced AC0D approach, fare for triplet excitation energies. The primary goal of this work is, however, to show how one can obtain accurate oscillator strengths for electronic transitions at different levels of AC-CAS theory. For this purpose, a first-order ERPA-based correction is proposed for the TDMs. Being able to compute the excitation energies of states with different symmetries and the corresponding oscillator strengths, the AC methods can become competitive with multireference approaches, such as CASPT2 and NEVPT2 as tools for simulating the electronic spectra of molecules.

In Section 2, we review the main ideas of the AC-based approaches for the correlation energy and propose to use intermediate quantities needed for the calculation of the AC0 energy to compute correlation-corrected TDMs and oscillator strengths. We investigate the performance of the AC, AC0, and AC0D methods for triplet excitations and for the oscillator strengths of organic molecules selected from ref (37) in Section 3. The work is summarized and summarized in Section 4.

## 2 Correlation-Corrected Oscillator Strengths from the Low-Order AC and Extended Random Phase Approximations

Given the reference wave functions for the ground and the Ith electronic states, and their corresponding energies, an excitation energy value and a 0 → *I* transition density matrix are immediately available; thus, a pertinent oscillator strength value can be computed. Its accuracy will be deficient due to a lack of accounting for dynamic correlation effects in its ingredients. In this section, we show that the AC approach combined with an ERPA provides a framework for computing correlation corrections for both transition energies and transition density matrices. For the convenience of the reader, we begin by briefly reviewing the AC and ERPA theories.

**Figure 1:**
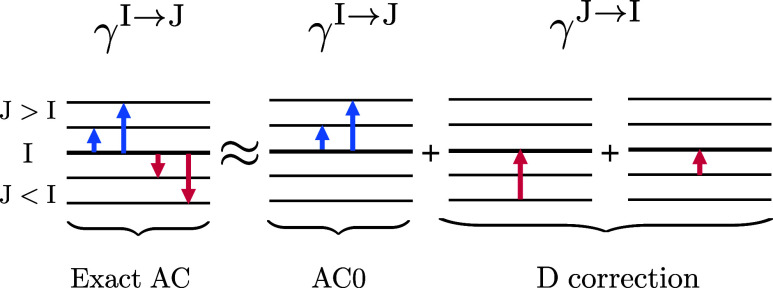
Terms included in the exact AC, see eq 7, the AC0 and AC0D approximations, cf. eq 20.

For an *I*th electronic state described with the Ψ_*I*_^ref^ reference wave function, the correlation energy is defined as a difference of exact and reference energies, namely
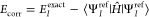
1AC formalism allows for finding correlation energy for any reference Ψ_*I*_^ref^.^38^ In ref (29), an AC approximation applicable for group product reference wave functions (e.g., CAS) has been proposed. General AC begins with choosing a Hamiltonian  for the reference wave function (for CAS reference, it can be a group product Hamiltonian^39^ or a Dyall^40^ Hamiltonian, both leading to the same working approximate AC equations^41^), and introducing an AC Hamiltonian
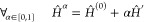
2which by varying the coupling constant α from 0 to 1 smoothly switches between a partially correlated system in the α = 0 limit (, Ψ_I_^α=0^ = Ψ_*I*_^ref^) and a fully correlated one at α = 1 (, Ψ_I_^α=1^ = Ψ_I_^exact^). Denote by Ψ_ν_^α^ and *E*_ν_^α^ eigenstates and energies of the AC Hamiltonian, . The correlation energy can be trivially expressed by the first-order derivative of the energy *E*_I_^α^
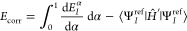
3Notice that expansion of the  derivative with respect to α around α = 0 formally leads to a perturbative series with the second-order correlation correction as its first term (the zeroth-order term  is canceled by )^41^
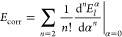
4In the AC formalism, however, one proceeds from eq 3 in a nonperturbative way by expressing two-electron reduced density matrix corresponding to the *I*th state of  by one-electron reduced properties, namely, one-electron reduced density matrix (1-RDM)
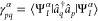
5and one-electron transition reduced density matrices (1-TRDM)
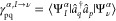
6Assuming that 1-RDM is fixed along the AC path leads to an approximate expression for the AC correlation energy reading^38,41^
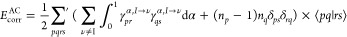
7where {*n*_*p*_} is a set of natural spin orbital occupation numbers and *pqrs* are indices of the natural spin orbitals, all pertaining the reference function Ψ_*I*_^ref^. ⟨*rs*|*pq*⟩ stands for a two-electron integral in the *x*_1_*x*_2_*x*_1_*x*_2_ convention. The prime sign in the sum indicates that the indices of the spin orbitals *pqrs* cannot simultaneously belong to the same group of orthogonal spin orbitals. The reference wave function Ψ_*I*_^ref^ in the remainder of this work will always be of CAS form. Notice that a sum-overstate summation with respect to ν runs over all states different from *I*, i.e. ,the states of the energies higher and lower than the *I*th state,^32^ see Figure 1.

The α-dependent 1-TRDMs can be obtained by employing Rowe’s equation of motion theory^42^ in the ERPA.^33,34^ Within that framework, excited states |Ψ_ν_⟩ are generated from an arbitrary state |Ψ⟩ upon the action of an excitation operator  which includes only single excitations
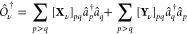
8For the α-dependent AC Hamiltonian, eq 2, matrices [**X**_ν_^α^] and [**Y**_ν_^α^] are eigenvectors of the α-dependent ERPA equations^33^ reading
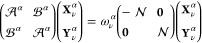
9where
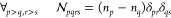
10The matrices  and  are defined as
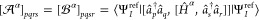
11They are linear in the coupling constant α
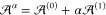
12similarly for , and presence of a double bracket in eq 12 leads to lowering the maximum rank of *n*-electron reduced density matrices, needed to evaluate the  and  matrices to only *n* = 2. Consequently, only 1- and 2-electron reduced density matrices are constructed from the reference wave function. The 1-TRDMs in the ERPA approximation read
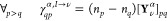
13
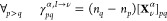
14and they are used in the evaluation of the AC correlation energy in eq 7.

### 2.1 Correlated Excitation Energies

While the above formulas are sufficient to obtain correlated energies of both ground (*I* = 0) and excited states (*I* > 0) for CAS self-consistent field (CASSCF) wave functions, the resulting approach, AC-CAS,^30,35^ can become prohibitively computationally expensive for large molecules and active spaces. An efficient algorithm avoiding the necessity to solve the expensive ERPA eigenproblem, eq 9, has been recently proposed.^36^ It is based on expanding the AC integrand, see eq 7, in order of the coupling constant α and computing corrections in each order from a recursive relation. In the lowest-order expansion, the AC integrand is linear in α and eq 7 leads to the AC0 approximation for the correlation energy^29,30^
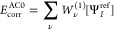
15The first-order contribution to the correlation energy from the νth state, *W*_ν_^(1)^, is expressed in a compact form by the zeroth-order eigenvectors {**Y**_ν_^(0)^, **X**_ν_^(0)^} and eigenvalues {ω_ν_^(0)^} of the ERPA eigenproblem and it reads
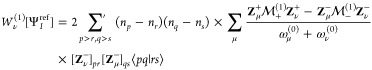
16where
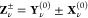
17The zeroth-order properties follow from solving the ERPA equations at α = 0. As it has been shown in ref (30), the ERPA matrices ,  are of a block-diagonal structure and diagonalization of the respective blocks scales with the fifth power of the system size. The  and  matrices in eq 16 are given by the positive and negative combinations of the correlation matrices, see eq 12
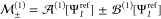
18

As discussed in ref (32), for the *I* > 0 excited state, the ERPA approximation is deficient as it neglects contributions of the lower-lying states to the density response function. This shortcoming leads to underestimating of the AC0 correlation energies computed for excited states and, consequently, to overestimating the corresponding excitation energies.^32^ In ref (32), we have used the property of 1-TRDM,  to show that the contribution to the correlation energy of a state *I* from a lower state *J* is the same as the contribution from state *I* to the correlation energy of state *J* (cf. Figure 1), namely
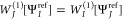
19Therefore, if  reference wave functions are available, the AC0-CAS excited-state correlation energy for the excited state *I* can be corrected at no additional computational cost by adding the missing “deexcitation” contributions *W*_*I*_^(1)^[Ψ_*J*_^ref^] for *J* < *I*, which gives rise to the AC0D approximation for the excited-state correlation energy
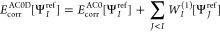
20We have previously shown that AC0D significantly improves the accuracy of singlet excitations comparing to AC0.^32^ In this work, we apply the AC0D method to triplet states. Application of the *D* correction (second term in the AC0D energy) for triplet states implies that summation with respect to *J* in eq 20 runs through triplet reference states.

To sum up, computation of an excitation energy for a transition 0 → *I* at the AC0D level employs the formula
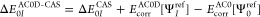
21and it requires an access to state-average CASSCF reference states from 0 up to *I*, finding the AC0 correlation energy from eq 15 for states 0 and *I* and adding the deexcitation correction for the excited state I.

### 2.2 Transition Dipole Moments

The oscillator strength of a transition from the ground state to the ν-th state defined as
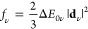
22involves the transition energy between the ground state and the ν-th excited state, Δ*E*_0ν_, and a corresponding TDM, **d**_ν_, given by 0 → ν 1-TRDM
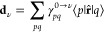
23The AC0 and ERPA approximations presented above allow one to predict the excitation energies and oscillator strengths at different theory levels. Beginning with the excitation energies, Δ*E*_0ν_, they can be computed using uncorrected CASS energies or from the correlated methods of an increasing level of accuracy, i.e., either AC0-CAS or AC0D-CAS; see eq 21.

Turning to transition density matrices **γ**^0→ν^ entering eq 23, they can be obtained directly from the reference CAS wave functions, Ψ_0_^ref^ and Ψ_ν_^ref^, leading to uncorrected CAS TDMs
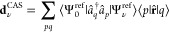
24The AC-ERPA equations, eqs 9–14, offer alternative approaches. To keep the computational cost at a low level and by relying on the near-linearity of the AC integrand,^38^ two ERPA approximations will be exploited: (1) the zeroth-order one, where transition density matrices are given by a pertinent zeroth-order ERPA eigenvectors corresponding to the 0 → ν transition
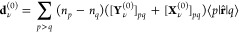
25and (2) **d**_ν_^(1)^, which assumes expansion of TRDMs from ERPA, see eqs 13 and 14, up to first-order in α
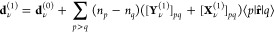
26The first-order corrections to ERPA eigenvectors **Y**_ν_^(1)^, **X**_ν_^(1)^ are obtained from a standard perturbation theory applied to the ERPA eigenproblem leading to the following expressions
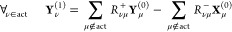
27
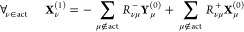
28where
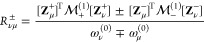
29and the coupling matrices  are defined in eq 18. Notice that eqs 27–29 pertain to states ν that are contained in the active space of orbitals. The νth eigenvectors and eigenvalues follow from diagonalization of the active block in ERPA equations at α = 0.^30^ Since ERPA is based on the excitation operator including singly excitations, eq 8, assignment of eigenvectors from ERPA in the 0th-order to CAS states should be straightforward for excited states of a single excitation character. For such states, the relations between zeroth-order ERPA eigenvectors and 1-TRDMs from CAS:  ≈ (*n_p_* − *n_q_*)[**Y**_ν_^(0)^]_*pq*_ and  ≈ (*n_q_* − *n_p_*)[**X**_ν_^(0)^]_*pq*_, and the zeroth-order eigenvalues and CAS transition energies, ω_ν_^(0)^ ≈ *E*_ν_^CAS^ – *E*_0_^CAS^, are closely satisfied. These relations have been used for state assignments for the molecules considered in this work.

Interestingly, correlation-corrected TDMs, eq 26, can also be computed for transitions to states of a double excitation character or in other cases when finding a correspondence between ERPA eigenvectors and CASSCF states is ambiguous. The formulas presented eqs 27–29 are useable as long as the 0 → ν transition density matrices and energies in the zeroth-order are available. One could then employ transition density matrix **γ**^CAS,0→ν^ and excitation energy *E*_ν_^CAS^ – *E*_0_^CAS^ from CASSCF calculation instead of, respectively, **Z**_ν_^(0)^ (using transformations given in eqs 13, 14 and 17) and ω_ν_^(0)^ quantities in the coupling matrix *R*_νμ_^±^ to obtain the first-order correlation correction for the zeroth-order CAS TDM. The zeroth-order TDMs, **d**_ν_^(0)^, contain no extra correlation compared to CAS-approximated ones, eq 24, and they should be of comparable accuracy. The quantities **d**_ν_^(1)^ given in eq 26 are anticipated to lead to oscillator strengths of superior accuracy as they are obtained by accounting for correlations of an in-active excitation ν with all out-of-active excitations. It is worth noticing that first-order transition density matrices are obtained as a byproduct of the evaluation of the AC0 energy and they do not involve extra computational costs.

## 3 Performance of the AC Methods for Electronically Excited States

### 3.1 Computational Details

We start by testing the performance of the AC0-CAS, AC0D-CAS, and ACD-CAS (which is simply an AC-CAS result with the deexcitation correction *D* added on top) methods on a set of triplet excitations of molecules taken from the benchmark set of Schreiber et al.^37^ of small organic compounds. Then, we use the same set of molecules to study the performance of the AC/ERPA methods for the oscillator strengths of singlet transitions.

Geometries of all molecules have been taken from ref (37). The CASSCF computations were performed in Molpro^43^ using the active spaces and the number of states in State-Average CASSCF (SA-CAS) from the work of Schreiber et al.^37^ except for *s*-tetrazine and benzene, see Table S1. The AC, AC0, and AC0D correlation corrections and AC-ERPA-based TDMs were computed in the GammCor program.^44^ All calculations have been performed in the TZVP basis set,^45^ apart from the basis state dependence study shown in the Supporting Information. We compare the excitation energies with CC3 and CASPT2 results from ref (37) and the oscillator strengths with CASPT2 results from ref (37) and with CC3 results from ref (46). The triplet excitation energies are also confronted with *n*-electron valence state perturbation theory (NEVPT2) results taken from ref (47).

### 3.2 Results and Discussion

**Figure 2:**
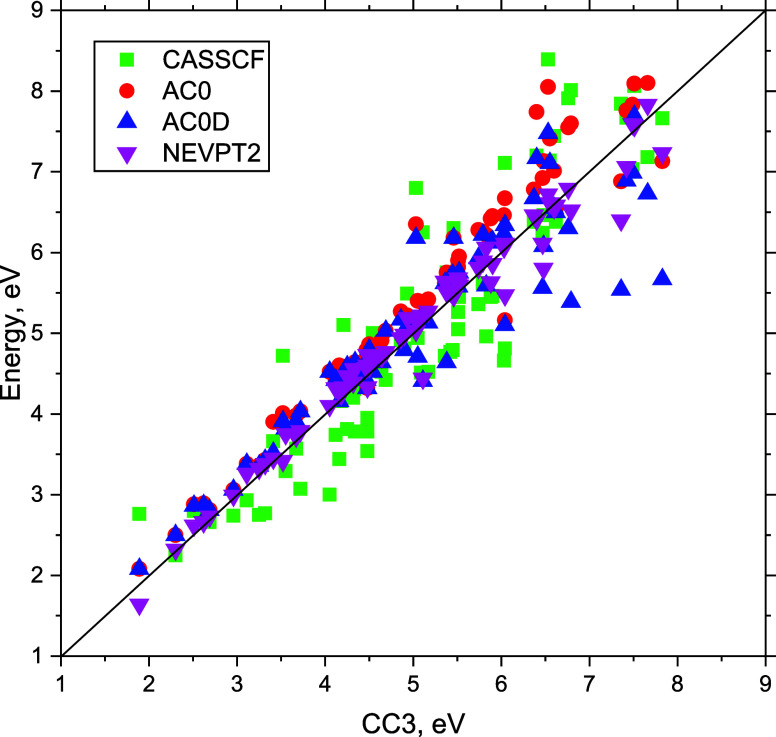
Triplet excitation energies in relation to the CC3 results. All values in eV.

Let us first look at the triplet excitation energies (see Figure 2, Tables 1 and S1). While the AC0 correction improves the accuracy of the CASSCF computation of the triplet excitation energies (the mean unsigned error, MUE, computed with respect to CC3 data, diminishes from 0.52 to 0.41 eV), it still tends to overestimate the higher state energies (see Figure 2, illustrated also by the value of mean error, ME, of 0.35 eV), similarly to the singlet ones.^32^ The de-excitation correction D partially alleviates this problem. The mean unsigned error (MUE) becomes smaller, 0.37 eV, but is still larger than the error of the NEVPT2 method (i.e., 0.27 eV). Clearly, see Figure 2, the largest errors of the AC0D method come from the higher excitations. The deexcitation correction in cases of benzene, naphthalene, pyridine, and *s*-tetrazine lowers the energies of some of the higher excited states to the point where the excitation energies are underestimated. This is true both for the AC0D and the ACD methods and consistent with the observations made in our previous work.^32^ The ERPA linear response function is an approximation deriving from the reference (in this case a state-averaged CASSCF) wave function. For higher-lying excited states, this approximation is frequently less accurate than for the ground state, which results in deteriorating accuracy of the excitation energies, which are calculated as differences of the excited- and ground-state energy.

**Table 1:** Mean Error (ME), MUE, and Standard Deviation (std. dev.) Evaluated with Respect to NEVPT2 and CC3 References for the Whole Set of Triplet Excitations and by Taking Two Lowest Excitations for Each Molecule (Numbers in Parentheses)^a^

	CASSCF	AC0	AC0D	ACD	CASPT2	CC3	NEVPT2
Errors with Respect to NEVPT2							
ME	–0.03 (−0.41)	0.33 (0.22)	0.00 (0.11)	–0.11 (−0.03)	–0.08 (−0.09)	–0.12 (−0.12)	
MUE	0.60 (0.55)	0.36 (0.23)	0.31 (0.17)	0.40 (0.29)	0.18 (0.10)	0.27 (0.12)	
std. dev.	0.74 (0.52)	0.36 (0.19)	0.45 (0.20)	0.53 (0.40)	0.33 (0.09)	0.24 (0.12)	
Errors with Respect to CC3							
ME	–0.02 (−0.34)	0.35 (0.29)	0.02 (0.19)	–0.08 (0.05)	–0.04 (−0.02)		0.12 (0.08)
MUE	0.52 (0.47)	0.41 (0.31)	0.37 (0.22)	0.40 (0.27)	0.08 (0.07)		0.27 (0.12)
std. dev.	0.67 (0.47)	0.35 (0.15)	0.55 (0.16)	0.60 (0.38)	0.11 (0.11)		0.24 (0.12)

a Notice that for ethene, only one excitation was taken into account. All values in eV.

For the set encompassing only the two lowest excitations for each molecule, the AC0D is significantly more accurate than AC0 (MUE of 0.22 eV vs 0.31 eV and ME of 0.19 eV vs 0.29 eV) and comparable to the NEVPT2 method (MUE of 0.12 eV and ME of 0.08 eV). Even for those selected excitations, the CASPT2 method is significantly more accurate (MUE = 0.07 eV). Interestingly, the more expensive method, ACD, does not perform better than the computationally efficient AC0D (MUE of 0.40 eV), except for the singlet–triplet gaps (see Supporting Information).

Notice that the underestimation of the excitation energies by the AC0D method is not systematic; while a few of the higher-lying excitations become significantly too low upon adding the deexcitation correction; others are almost perfectly aligned with the NEVPT2 results (cf. Figure 2). The fact that the errors produced by the ACD method are very similar to the ones in AC0D proves that the source of inaccuracy does not lie in the AC0 approximation. Further investigations into this problem are warranted.

Let us now look at the performance of the AC-based methods for the oscillator strengths. It is clear from eq 22 that for obtaining accurate oscillator strengths, both good-quality excitation energies and TDMs are necessary. Even though it is possible to obtain the TDMs at CASPT2 level,^48,49^ in the case of perturbation-theory computations, the oscillator strengths are often computed using the CASPT2 excitation energies and CASSCF TDMs. It is therefore interesting to check the influence of both the excitation energy and TDM on the overall accuracy of the oscillator strengths. We compare the results to oscillator strengths calculated at the linear-response CC3 (LR-CC3)/TZVP level taken from ref (46). Higher-level theory benchmarks are available in the Dunning basis sets^50,51^ for some of the molecules from the Schreiber et al.^37^ test set, but the TDMs are quite sensitive to the basis set choice,^52^ so they were not suitable for this comparison. At the same time, LR-CC3 was shown to be an accurate and reliable method of calculating the oscillator strengths.^46^

In Table 2, we see the oscillator strengths of the bright states of the studied molecules calculated using combinations of approximations for the excitation energies and the TDMs. For the former, the CASSCF, AC0, and AC0D excitation energies have been employed, and for the latter, the uncorrelated approximations **d**_ν_^CAS^ and **d**_ν_^(0)^ calculated according to eqs 24 and 25, and the first-order-corrected **d**_ν_^(1)^ expression, cf. eq 26, have been used. Notice that for the formamide molecule, we assigned states in agreement with the newer works of Kánnár and Szalay^46^ and Helmich-Paris^53^ rather than the seminal work of Schreiber et al.^37^

**Table 2:** Oscillator Strengths for Singlet Excitations in Atomic Units^a^

		**d**_ν_^CAS^			**d**_ν_^(0)^		**d**_ν_^(1)^			
molecule	state	CASSCF	AC0	AC0D	AC0	AC0D	AC0	AC0D	CASPT2	CC3
ethene	1 ^1^*B*_1*u*_	0.576	0.604	0.567	0.604	0.567	0.450	0.422	0.513	0.389
*E*-butadiene	1 ^1^*B*_1*u*_	1.071	1.009	0.897	1.005	0.893	0.827	0.735	0.783	0.726
all-*E*-hexatriene^1^	1 ^1^*B*_1*u*_	1.365	1.451	1.332	1.442	1.325	1.303	1.197	1.045	1.129
all-*E*-octatetraene^1^	1 ^1^*B*_1*u*_	1.622	1.728	1.605	1.719	1.597	1.631	1.515	1.299	1.549
cyclopropene	1 ^1^*B*_1_	0.009	0.009	0.009	0.009	0.009	0.001	0.001	0.010	0.001
	1 ^1^*B*_2_	0.322	0.326	0.277	0.326	0.277	0.083	0.071	0.234	0.083
cyclopentadiene	1 ^1^*B*_2_	0.219	0.199	0.173	0.195	0.169	0.078	0.068	0.144	0.093
	2 ^1^*A*_1_	0.000	0.000	0.000	0.002	0.002	0.000	0.000	0.001	0.005
norbornadiene	1 ^1^*B*_2_	0.143	0.126	0.112	0.131	0.117	0.031	0.028	0.092	0.027
benzene	1 ^1^*B*_2*u*_	0.000	0.000	0.000	0.000	0.000	0.000	0.000	0.001	0.000
	2 ^1^*E*_1*u*_	1.115	1.086	0.912	1.060	0.891	0.724	0.608	0.847	0.630
naphthalene	1 ^1^*B*_3*u*_	0.000	0.000	0.000	0.000	0.000	0.000	0.000	0.001	0.000
	1 ^1^*B*_2*u*_	0.100	0.088	0.073	0.122	0.101	0.066	0.055	0.137	0.085
	2 ^1^*B*_3*u*_	0.022	0.024	0.018	2.089	1.536	1.708	1.256	1.548	1.325
	2 ^1^*B*_2*u*_	0.351	0.342	0.245	0.586	0.419	0.349	0.250	0.402	0.239
furan	1 ^1^*B*_2_	0.228	0.217	0.190	0.255	0.224	0.138	0.121	0.199	0.155
	2 ^1^*A*_1_	0.000	0.000	0.000	0.000	0.000	0.005	0.004	0.008	0.001
	3 ^1^*A*_1_	0.764	0.832	0.716	1.092	0.940	0.574	0.494	0.793	0.450
pyrrole	2 ^1^*A*_1_	0.001	0.001	0.001	0.000	0.000	0.000	0.000	0.031	0.004
	1 ^1^*B*_2_	0.130	0.131	0.114	0.209	0.184	0.124	0.108	0.205	0.167
	3 ^1^*A*_1_	0.836	0.899	0.790	1.030	0.906	0.588	0.517	0.613	0.478
imidazole	2 ^1^*A*′	0.009	0.010	0.009	0.257	0.240	0.157	0.147	0.229	0.081
	3 ^1^*A*′	0.195	0.198	0.182	0.002	0.002	0.004	0.004	0.062	0.082
	1 ^1^*A*″	0.044	0.046	0.045	0.023	0.023	0.004	0.004	0.010	0.004
pyridine^1^	1 ^1^*B*_2_	0.009	0.010	0.010	0.035	0.034	0.025	0.024	0.044	0.021
	2 ^1^*A*_1_	0.007	0.007	0.006	0.047	0.041	0.041	0.035	0.004	0.014
	3 ^1^*A*_1_	0.928	0.999	0.651	0.908	0.700	0.565	0.435	0.849	0.526
	2 ^1^*B*_2_	0.866	0.956	0.758	0.912	0.818	0.587	0.526	0.691	0.482
pyrazine	1 ^1^*B*_3*u*_	0.016	0.016	0.016	0.020	0.020	0.005	0.005	0.012	0.007
	1 ^1^*B*_2*u*_	0.036	0.041	0.039	0.122	0.115	0.082	0.077	0.123	0.062
	1 ^1^*B*_1*u*_	0.077	0.076	0.063	0.156	0.129	0.131	0.108	0.107	0.070
	2 ^1^*B*_1*u*_	1.025	0.999	0.651	0.881	0.574	0.442	0.288	0.774	0.407
	2 ^1^*B*_2*u*_	0.964	0.956	0.758	0.784	0.621	0.458	0.363	0.622	0.376
pyrimidine	1 ^1^*B*_1_	0.016	0.016	0.016	0.024	0.024	0.006	0.006	0.013	0.006
	1 ^1^*B*_2_	0.010	0.011	0.011	0.042	0.041	0.027	0.026	0.049	0.021
	2 ^1^*A*_1_	0.010	0.010	0.009	0.080	0.068	0.058	0.049	0.164	0.043
pyridazine	1 ^1^*B*_1_	0.010	0.009	0.009	0.015	0.014	0.005	0.005	0.010	0.001
	2 ^1^*A*_1_	0.009	0.010	0.009	0.027	0.025	0.016	0.015	0.027	0.014
*s*-tetrazine	1 ^1^*B*_3*u*_	0.022	0.016	0.016	0.023	0.023	0.007	0.007	0.013	0.007
	1 ^1^*B*_2*u*_	0.037	0.039	0.037	0.127	0.119	0.077	0.072	0.110	0.044
	2 ^1^*B*_3*u*_	0.007	0.006	0.006	0.007	0.005	0.012	0.010	0.021	0.011
	1 ^1^*B*_1*u*_	0.033	0.029	0.027	0.002	0.001	0.000	0.000	0.136	0.002
	2 ^1^*B*_1*u*_	0.554	0.537	0.438	0.456	0.435	0.334	0.319	0.496	0.349
formaldehyde	1 ^1^*B*_1_	0.009	0.009	0.009	0.009	0.009	0.003	0.003	0.013	0.003
	2 ^1^*A*_1_	0.492	0.517	0.438	0.502	0.425	0.208	0.176	0.451	0.348
acetone	1 ^1^*B*_1_	0.005	0.006	0.006	0.006	0.006	0.001	0.001	0.011	0.000
	2 ^1^*A*_1_	0.590	0.617	0.523	0.617	0.523	0.320	0.271	0.358	0.245
*p*-benzoquinone	1 ^1^*B*_1*u*_	0.543	0.537	0.398	0.909	0.675	0.595	0.442	0.638	0.485
formamide	2 ^1^*A*′	0.550	0.581	0.506	0.497	0.433	0.273	0.238	0.479	0.386
	3 ^1^*A*′	0.204	0.215	0.193	0.294	0.265	0.067	0.060	0.163	0.110
propanamide	2 ^1^*A*′	0.482	0.505	0.453	0.420	0.376	0.211	0.189	0.405	0.136
	3 ^1^*A*′	0.337	0.354	0.318	0.452	0.407	0.242	0.218	0.275	0.170
acetamide	1 ^1^*A*″	0.001	0.001	0.001	0.001	0.001	0.000	0.000	0.001	0.001
	2 ^1^*A*′	0.500	0.525	0.470	0.439	0.392	0.219	0.196	0.424	0.207
	3 ^1^*A*′	0.326	0.344	0.310	0.442	0.398	0.277	0.250	0.263	0.263

a Labels CASSCF, AC0, and AC0D and **d**_ν_^CAS^, **d**_ν_^(0)^, **d**_ν_^(1)^ denote, respectively, methods used for computation of excitation energies and TDMs. ^1^In the CASSCF calculation, the TDMs for 1 ^1^*B*_1*u*_ states in all-*E*-hexatriene and all-*E*-octatetraene and for 3 ^1^*A*_1_ and 2 ^1^*B*_2_ states in pyridine were switched with higher lying states of the same symmetry. For a fair comparison between methods, we used the correct CASSCF TDMs.

**Figure 3:**
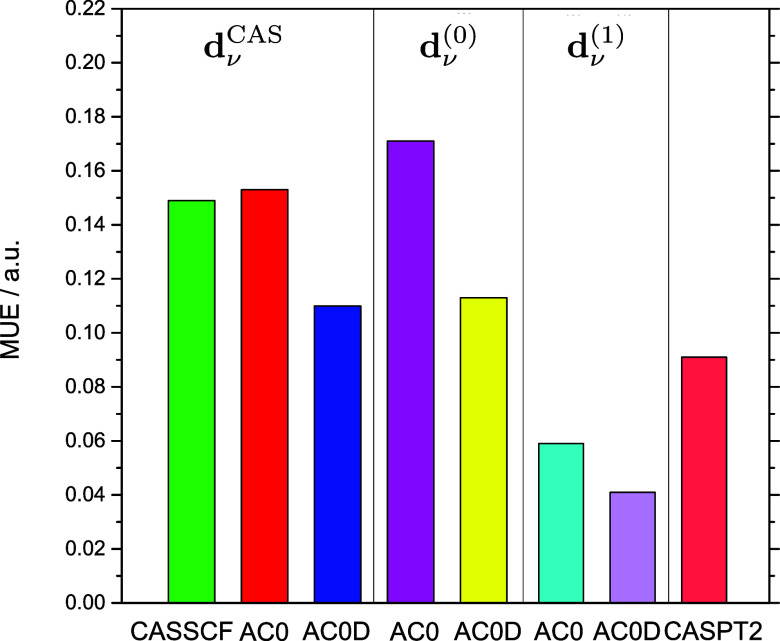
MUE of oscillator strengths in reference to CC3 values.

The results presented in Table 3 and Figure 3 show that the oscillator strengths obtained at the CASSCF level are not improved if excitation energies are corrected by AC0 correlation energy. Adding the *D* correction and describing transition energies by the AC0D method leads to improvements in oscillator strengths both when the oscillator strengths are described by the CASSCF method (AC0D/**d**_ν_^CAS^) or by the ERPA (AC0D/**d**_ν_^(0)^) by 0.039 au of AC0D/**d**_ν_^CAS^ and by 0.036 au of AC0D/**d**_ν_^CAS^ with respect to the pure CASSCF approach.

Only when the TDMs are corrected in the first-order, **d**_ν_^(1)^, do we see a significant improvement in the results. Adding a first-order correction reduces the MUE of the oscillator strengths by more than half with respect to the ones calculated with uncorrected ERPA dipole moments **d**_ν_^(0)^, i.e., the MUE of AC0/**d**_ν_^(1)^ is 0.059 au vs 0.171 au for AC0/**d**_ν_^(0)^ and for AC0D/**d**_ν_^(1)^, it is 0.041 au vs 0.113 au for AC0D/**d**_ν_^(0)^. Similarly, other dispersion measures such as the mean error and standard deviation present the AC0 and AC0D computations with corrected TDMs as reliable methods of obtaining the oscillator strengths. Interestingly, the AC0/**d**_ν_^(1)^ and AC0D/**d**_ν_^(1)^ produce even smaller MUEs with respect to LR-CC3 than CASPT2 does, i.e., 0.091 au One may wonder if LR-CC3 is a better benchmark than CASPT2. The LR-CC3 method’s accuracy for oscillator strengths has been indeed validated by multiple works.^46,50^ Unlike CASPT2, LR-CC3 is largely a “black box” type method that minimizes the risk of obtaining erratic or wrongly assigned results. The agreement between LR-CC3, AC0/**d**_ν_^(1)^, and AC0D/**d**_ν_^(1)^ results is therefore a good indicator of the accuracy and stability of the latter two methods.

The largest errors in the oscillator strengths obtained by AC0 and AC0D methods correspond to higher states of the molecules such as naphthalene or *s*-tetrazine (cf. Table 2). This is related to the poorer description of the higher excited states in the AC/ERPA approximation which we explained in our previous work.^32^

**Figure 4:**
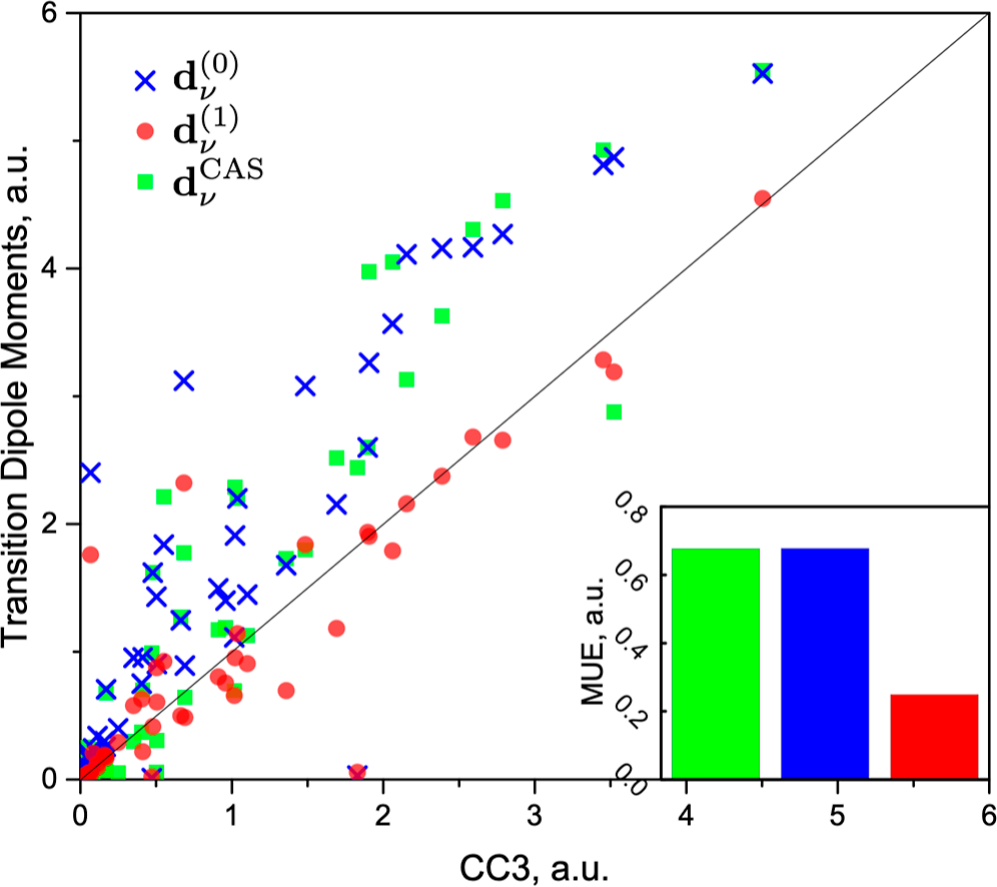
TDMs **d**_ν_^(0)^, **d**_ν_^(1)^, and **d**_ν_^CAS^ for single excitations of molecules vs TDMs computed with CC3 method. Inset: mean unsigned errors of TDMs w.r.t. CC3 values.

To separate the effect of improved accuracy of the excitation energies calculation from the TDM computations, we also confronted the values of TDMs **d**_ν_^(0)^, **d**_ν_^(1)^, and **d**_ν_^CAS^ with the CC3 values (see Figure 4). The correlation plot and the MUE show that the TDMs from a CASSCF computation, **d**_ν_^CAS^, and uncorrected ERPA TDMs, **d**_ν_^(0)^, not only have similar quality but also are quite close to each other in value. Both methods tend to overestimate the TDMs. On the other hand, the AC0D method of computing the excitation energies tends to underestimate energies of the higher-lying excited states (cf. Figure 2 and ref (32)). One might therefore suspect a cancellation of errors effect when AC0D excitation energies and either **d**_ν_^CAS^ or **d**_ν_^(0)^ TDMs are combined to compute the oscillator strengths (cf. eq 22). From Table 3, it is however clear that this effect is quite subtle. The MUE values of AC0D/**d**_ν_^CAS^ and AC0D/**d**_ν_^(0)^ are about 30% smaller than correspondingly MUEs of AC0D/**d**_ν_^CAS^ and AC0D/**d**_ν_^(0)^ values. The corrected TDMs, **d**_ν_^(1)^, paint a different picture. Not only is the MUE cut by two-thirds with respect to the **d**_ν_^(0)^ and **d**_ν_^CAS^ TDMs but also the systematic overestimation is no longer a problem. Hence, we find that the improvement of the oscillator strengths when **d**_ν_^(1)^ are used is not a result of the error cancellation but of the better accuracy of the used TDMs.

**Table 3:** Mean Error (ME), MUE, and Standard Deviation (std. dev.) Evaluated with Respect to CASPT2 and CC3 References for the Whole Set of Oscillator Strengths^a^

	**d**_ν_^CAS^			**d**_ν_^(0)^		**d**_ν_^(1)^		
	CASSCF	AC0	AC0D	AC0	AC0D	AC0	AC0D	CASPT2
Errors with Respect to CC3								
ME	0.097	0.105	0.056	0.163	0.103	0.031	–0.008	0.162
MUE	0.153	0.161	0.118	0.164	0.107	0.053	0.035	0.100
std. dev.	0.263	0.270	0.230	0.197	0.128	0.091	0.067	0.127
Errors with Respect to CASPT2								
ME	0.015	0.024	–0.026	0.081	0.021	–0.051	–0.090	
MUE	0.107	0.115	0.094	0.093	0.056	0.080	0.100	
std. dev.	0.243	0.250	0.234	0.138	0.093	0.112	0.124	

a Labels CASSCF, AC0, and AC0D and **d**_ν_^CAS^, **d**_ν_^(0)^, **d**_ν_^(1)^ denote, respectively, methods used for computation of excitation energies and TDMs.

For a broader picture of the performance of the discussed approaches, we show the basis set dependence of the results for selected systems in Table S3 of the Supporting Information and compare them to the benchmarks available for those basis sets. As expected, oscillator strengths obtained from the ERPA TDMs show a somewhat stronger basis set dependence than the ones obtained with CASSCF TDMs. For those selected systems, the oscillator strengths using the corrected TDMs, **d**_ν_^(1)^, come much closer than the ones deriving from CASSCF or the uncorrected ERPA TDMs to the CCSD and FCI benchmark values in the corresponding basis sets, which is in agreement with conclusions obtained for the Schreiber et al.^37^ data set.

## 4 Summary and Conclusions

Continuing our work in ref (32), we have presented the performance of the AC0D method for triplet excitation energies on the test set of Schreiber et al.^37^ We showed that the deexcitation correction indeed improves the performance of the AC0 method, in particular, when applied to the lowest excited states. For those states, its accuracy is comparable to the accuracy of LR-CC2 and LR-CCSD methods.^37^ Notice, however, that unlike LR-CC2 and LR-CCSD, the AC0 and AC0D methods are not limited to single-excitation-dominated excited states.

We also presented two new AC/ERPA-based approaches to calculate the oscillator strengths of electronic excitations. The first one employs zeroth-order transition reduced density matrices obtained from the ERPA calculation without additional cost. It has an accuracy comparable to that of the CASSCF calculation performed in the same active space. The second one, utilizing first-order corrections to the ERPA transition density matrices, has excellent accuracy, similar to or better than that of the CASPT2 method, attained at a lower computational cost. What is more, there is no additional cost to calculating the oscillator strengths when AC0 or AC0D excitation energy calculations are performed.

We have shown that the AC ERPA-based methods such as AC0 and AC0D are capable of describing electronic excitations and oscillator strengths of molecules. Good accuracy of the oscillator strengths is owed to corrected ERPA transition density matrices and AC0D excitation energies. Notice that the former have been already successfully exploited in multireference symmetry adapted perturbation theory to improve dispersion and induction molecular interaction energy terms.^54−56^ In the future, they can be used to obtain other properties.

## Supporting Information Available

Total energies, triplet excitation energies, singlet–triplet gaps of studied molecules, oscillator strengths calculated in augmented double-, triplet-, and quadruple-ζ Dunning basis sets, and errors of computed singlet–triplet gaps (PDF)
