# Supplementary material for: Multireference Correlated Oscillator Strengths from Adiabatic Connection Approaches Based on Extended Random Phase Approximation
Source: J Chem Theory Comput. 2024 Apr 26;20(9):3659–68. doi: 10.1021/acs.jctc.4c00103 (PMC11099974; doi:10.1021/acs.jctc.4c00103)
Supplement: Supplementary file 1 — ct4c00103_si_001.pdf [file ct4c00103_si_001.pdf]

Supplementary Material for "Multireference Correlated Oscillator Strengths from Adiabatic Connection Approach Based on Extended Random Phase Approximation"

Daria Drwal,[1] Katarzyna Pernal,[1] and Ewa Pastorczak[1]

[1]*Institute of Physics, Lodz University of Technology,*
ul. Wolczanska 219, 90-924 Lodz, Poland

I. TRIPLET EXCITATION ENERGIES

TABLE S1: Vertical triplet excitation energies in eV. CASPT2, CC3 were taken from Ref. 1 and NEVPT2 results were taken from Ref. 2

Molecule	State	CASSCF	AC0	AC0D	ACD	CASPT2	CC3	NEVPT2
Ethene	$1\,^3B_{1u}$	3.78	4.54	4.54	4.48	4.60	4.48	4.60
E-butadiene	$1\,^3B_{1u}$	2.77	3.43	3.43	3.22	3.34	3.32	3.38
	$1\,^3A_g$	4.52	5.42	5.13	4.83	5.16	5.17	5.27
all-*E*-hexatriene	$1\,^3B_{1u}$	2.66	2.81	2.81	2.78	2.71	2.69	2.73
	$1\,^3A_g$	4.20	4.56	4.50	4.39	4.31	4.32	4.39
all-*E*-octatetraene	$1\,^3B_{1u}$	2.25	2.50	2.50	2.41	2.33	2.30	2.32
	$1\,^3A_g$	3.57	3.98	3.91	3.76	3.70	3.67	3.72
Cyclopropene	$1\,^3B_1$	6.38	6.54	6.50	6.76	6.51	6.62	6.58
	$1\,^3B_2$	3.78	4.64	4.64	4.61	4.35	4.34	4.56
Cyclopentadiene	$1\,^3B_2$	2.75	3.36	3.36	3.15	3.28	3.25	3.32
	$1\,^3B_1$	4.51	5.39	5.20	4.87	5.10	5.09	5.22
Norbornadiene	$1\,^3A_2$	3.07	4.03	4.03	3.70	3.75	3.72	3.79
	$1\,^3B_2$	3.44	4.6	4.16	3.73	4.22	4.16	4.30
Benzene[a]	$1\,^3B_{1u}$	3.74	4.42	4.42	5.25	4.17	4.12	4.32
	$1\,^3E_{1u}$	4.81	5.20	4.79	6.07	4.90	4.90	4.98
	$1\,^3B_{2u}$	4.81	5.16	5.10	4.36	5.76	6.04	5.47
	$2\,^3E_{2g}$	7.03	7.83	6.99	6.92	7.41	7.49	7.59
Naphthalene	$1\,^3B_{2u}$	2.93	3.38	3.38	3.21	3.20	3.11	3.26
	$1\,^3B_{3u}$	4.16	4.46	4.44	4.34	4.29	4.18	4.24
	$2\,^3B_{1g}$	4.33	4.79	4.42	4.27	4.55	4.47	4.57
	$2\,^3B_{2u}$	4.52	4.91	4.64	4.50	4.71	4.64	4.70
	$2\,^3B_{3u}$	6.25	5.39	4.41	4.65	5.00	5.11	4.44
	$1\,^3A_g$	5.45	5.95	5.75	5.58	5.57	5.52	5.59

Molecule	State	CASSCF	AC0	AC0D	ACD	CASPT2	CC3	NEVPT2
	$2\,^3B_{1g}$	6.46	7.13	6.08	5.86	6.25	6.48	5.80
	$2\,^3A_g$	6.24	6.92	5.56	5.34	6.42	6.47	6.11
	$3\,^3A_g$	8.01	7.60	5.39	5.52	6.63	6.79	6.52
	$3\,^3B_{1g}$	7.91	7.55	6.30	6.45	6.67	6.76	6.79
Furan	$1\,^3B_2$	3.54	4.32	4.32	4.06	4.17	4.48	4.33
	$1\,^3A_1$	5.05	5.82	5.58	5.31	5.49	5.51	5.62
Pyrrole	$1\,^3B_2$	3.95	4.68	4.68	4.44	4.52	4.48	4.73
	$1\,^3A_1$	5.26	5.90	5.58	5.35	5.53	5.51	5.68
Imidazole	$1\,^3A'$	4.42	5.03	5.03	4.88	4.65	4.69	4.77
	$2\,^3A'$	5.62	6.26	6.22	6.20	5.74	5.79	5.89
	$1\,^3A''$	6.38	6.78	6.67	6.53	6.36	6.37	6.46
	$3\,^3A'$	7.14	7.41	7.11	6.96	6.44	6.55	6.61
	$4\,^3A'$	7.67	7.76	6.89	6.84	7.43	7.42	7.06
	$2\,^3A''$	8.06	8.09	7.69	7.67	7.51	7.51	7.57
Pyridine	$1\,^3A_1$	3.81	4.59	4.59	4.34	4.27	4.25	4.47
	$1\,^3B_2$	4.91	5.27	5.17	5.10	4.72	4.86	4.94
	$2\,^3A_1$	4.94	5.40	4.71	4.55	5.03	5.05	5.13
	$2\,^3B_2$	7.20	7.74	7.17	7.01	6.02	6.40	6.41
	$3\,^3A_1$	7.18	8.10	6.73	6.45	7.56	7.66	7.83
	$3\,^3B_2$	7.66	7.13	5.67	5.77	7.88	7.83	7.23
	$1\,^3B_1$	4.82	4.86	4.80	4.78	4.55	4.50	4.58
	$1\,^3A_2$	6.30	6.18	6.18	6.22	5.48	5.46	5.46
s-tetrazine[b]	$1\,^3B_{3u}$	2.76	2.08	2.08	2.24	1.56	1.89	1.64
	$1\,^3A_u$	4.72	4.01	3.91	4.03	3.26	3.52	3.42
	$1\,^3B_{1g}$	5.10	4.50	4.38	4.52	4.14	4.21	4.33
	$1\,^3B_{1u}$	4.31	4.55	4.47	4.35	4.36	4.33	4.55
	$1\,^3B_{2u}$	5.00	4.76	4.52	4.57	4.56	4.54	4.72

Molecule	State	CASSCF	AC0	AC0D	ACD	CASPT2	CC3	NEVPT2
	$1\,^3B_{2g}$	5.49	5.22	5.09	5.14	4.93	4.93	5.19
	$2\,^3A_u$	6.80	6.35	6.18	6.32	5.02	5.03	5.03
	$1\,^3B_{3g}$	7.62	7.38	6.73	6.51	5.50		7.81
	$2\,^3B_{1u}$	5.75	5.75	4.64	4.58	5.40	5.38	5.51
	$2\,^3B_{2g}$	7.11	6.67	6.34	6.43	5.97	6.04	6.11
	$2\,^3B_{1g}$	7.44	7.01	6.55	6.66	6.31	6.60	6.55
	$2\,^3B_{3u}$	8.39	8.05	7.48	7.54	6.54	6.53	6.72
Formaldehyde	$1\,^3A_2$	3.32	3.80	3.80	3.49	3.75	3.55	3.58
	$1\,^3A_1$	4.99	6.19	5.57	5.01	5.84	5.83	6.06
Acetone	$2\,^1A_1$	3.00	4.52	4.52	4.14	4.08	4.05	4.10
	$2\,^1A_2$	4.66	6.46	6.23	5.82	6.03	6.03	6.06
p-benzoquinone	$1\,^3B_{1g}$	2.80	2.88	2.86	2.71	2.63	2.51	2.62
	$1\,^3B_{Au}$	2.80	2.89	2.87	2.70	2.68	2.62	2.66
	$1\,^3B_{1u}$	2.74	3.06	3.06	2.92	2.99	2.96	2.99
	$1\,^3B_{3g}$	3.66	3.90	3.52	3.39	3.31	3.41	3.45
Formamide	$1\,^3A''$	4.72	5.62	5.62	5.59	5.40	5.36	5.64
	$1\,^3A'$	5.36	6.28	5.93	5.15	5.58	5.74	5.81
Acetamide	$1\,^3A''$	4.77	5.69	5.69	5.39	5.53	5.42	5.52
	$1\,^3A'$	5.45	6.42	6.12	5.78	5.75	5.88	5.63
Propanamide	$1\,^3A''$	4.79	5.72	5.72	5.73	5.44	5.45	5.54
	$1\,^3A'$	5.46	6.45	6.14	5.67	5.79	5.90	5.86

[a] active space (0 0 0 0 2 1 2 1)6, in parenthesis number of active orbitals in respective symmetries: A_g B_{3u} B_{2u} B_{1g} B_{1u} B_{2g} B_{3g} A_u

[b] active space (2 2 2 1 2 2 2 1)14, in parenthesis number of active orbitals in respective symmetries: A_g B_{3u} B_{2u} B_{1g} B_{1u} B_{2g} B_{3g} A_u

II. TOTAL ENERGIES

Molecule	State	CASSCF	AC0	AC0D	AC
Ethene	$1\,^1A_g$	-78.073279	-78.420381	-78.420381	-78.418412
	$1\,^3B_{1u}$	-77.934370	-78.253607	-78.253607	-78.253639
E-butadiene	$1\,^1A_g$	-155.010171	-155.685995	-155.685995	-155.661878
	$1\,^3B_u$	-154.908405	-155.559881	-155.559881	-155.543577
	$2\,^3A_g$	-154.844141	-155.486758	-155.497288	-155.473615
all-E-hexatriene	$1\,^1A_g$	-231.968532	-232.949336	-232.949336	-232.918796
	$2\,^3A_g$	-231.814175	-232.781936	-232.784031	-232.816566
	$1\,^3B_u$	-231.870906	-232.845917	-232.845917	-232.755393
all-E-octatetraene	$1\,^1A_g$	-308.908761	-310.214806	-310.214806	-310.16982
	$1\,^3B_u$	-308.826188	-310.122942	-310.122942	-310.081164
	$1\,^3A_g$	-308.777559	-310.068591	-310.071240	-310.029106
Cyclopropene	$1\,^1A_g$	-115.869031	-116.393825	-116.393825	-116.376504
	$1\,^3B_1$	-115.634651	-116.153607	-116.154816	-116.128161
	$1\,^3B_2$	-115.730163	-116.223351	-116.223351	-116.206955
Cyclopentadiene	$1\,^1A_g$	-192.893743	-193.737881	-193.737881	-193.700145
	$1\,^3B_2$	-192.792821	-193.614523	-193.614523	-193.584485
	$2\,^3A_1$	-192.728171	-193.539708	-193.546786	-193.514163
Norbornadiene	$1\,^1A_g$	-269.771008	-270.991368	-270.991368	-270.925955
	$1\,^3A_2$	-269.658356	-270.843226	-270.843226	-270.789876
	$1\,^3B_2$	-269.644433	-270.822331	-270.838485	-270.772703
Benzene	$1\,^1A_g$	-230.847041	-231.820853	-231.820853	-231.786916
	$1\,^3B_{2u}$	-230.709691	-231.658267	-231.658267	-231.594161
	$2\,^3B_{2u}$	-230.670344	-231.629734	-231.644959	-231.548955
	$1\,^3B_{3u}$	-230.670403	-231.631118	-231.633475	-231.624485
	$1\,^3A_g$	-230.588596	-231.533023	-231.563880	-231.501595
Naphthalene	$1\,^1A_g$	-383.596089	-385.201027	-385.201027	-385.126495

Molecule	State	CASSCF	AC0	AC0D	AC
	$1\,^3B_{2u}$	-383.488540	-385.076906	-385.076906	-385.008435
	$1\,^3B_{3u}$	-383.443100	-385.037021	-385.037718	-384.966148
	$1\,^3B_{1g}$	-383.437071	-385.024893	-385.038449	-384.956084
	$2\,^3B_{2u}$	-383.430061	-385.020724	-385.030682	-384.951266
	$2\,^3B_{3u}$	-383.366507	-385.002955	-385.039122	-384.919560
	$1\,^3A_g$	-383.395898	-384.98226	-384.98981	-384.914052
	$2\,^3B_{1g}$	-383.358605	-384.938827	-384.977446	-384.872554
	$2\,^3A_g$	-383.366769	-384.946586	-384.996696	-384.880349
	$3\,^3A_g$	-383.301719	-384.921888	-385.002889	-384.842540
	$3\,^3B_{1g}$	-383.305460	-384.923483	-384.969652	-384.843401
Furan	$1\,^1A_g$	-228.749232	-229.660864	-229.660864	-229.600620
	$1\,^3B_2$	-228.618961	-229.502002	-229.502002	-229.451437
	$1\,^3A_1$	-228.563733	-229.447063	-229.455929	-229.396833
Pyrrole	$1\,^1A_g$	-208.923732	-209.812417	-209.812417	-209.760011
	$1\,^3B_2$	-208.778730	-209.640531	-209.640531	-209.597006
	$1\,^3A_1$	-208.730576	-209.595445	-209.607313	-209.551652
Imidazole	$1\,^1A_g$	-224.951407	-225.859536	-225.859536	-225.800891
	$1\,^3A'$	-224.78899	-225.674714	-225.674714	-225.621704
	$2\,^3A'$	-224.74473	-225.629513	-225.630809	-225.571739
	$1\,^3A''$	-224.7168	-225.610293	-225.614280	-225.556695
	$3\,^3A'$	-224.689168	-225.587176	-225.598113	-225.534173
	$4\,^3A'$	-224.669696	-225.574362	-225.606398	-225.517372
	$2\,^3A''$	-224.655387	-225.562401	-225.566920	-225.504434
Pyridine	$1\,^1A_g$	-246.850209	-247.853581	-247.853581	-247.803313
	$1\,^3A_1$	-246.710124	-247.685055	-247.685055	-247.644000
	$1\,^3B_2$	-246.669691	-247.659755	-247.663472	-247.612128
	$2\,^3A_1$	-246.668492	-247.655081	-247.680391	-247.610585

Molecule	State	CASSCF	AC0	AC0D	AC
	$2\,^3B_2$	-246.585764	-247.569310	-247.590109	-247.524813
	$3\,^3A_1$	-246.586461	-247.555914	-247.606391	-247.515792
	$3\,^3B_2$	-246.568555	-247.591613	-247.645102	-247.537559
	$1\,^3B_1$	-246.673233	-247.675060	-247.677033	-247.625344
	$1\,^3A_2$	-246.618742	-247.626416	-247.626577	-247.574839
s-Tetrazine	$1\,^1A_g$	-294.789725	-295.880493	-295.880493	-295.828771
	$1\,^3B_{3u}$	-294.698524	-295.805830	-295.805830	-295.746501
	$1\,^3A_u$	-294.631533	-295.731357	-295.735506	-295.677067
	$1\,^3B_{1g}$	-294.618443	-295.715442	-295.719254	-295.658307
	$1\,^3B_{1u}$	-294.654544	-295.718493	-295.724409	-295.665852
	$1\,^3B_{2u}$	-294.618708	-295.700548	-295.714995	-295.652187
	$1\,^3B_{2g}$	-294.607166	-295.682647	-295.695023	-295.635005
	$2\,^3A_u$	-294.565717	-295.649388	-295.656446	-295.590390
	$1\,^3B_{3g}$	-294.533755	-295.583664	-295.630326	-295.565710
	$2\,^3B_{1u}$	-294.595600	-295.666020	-295.724931	-295.619573
	$2\,^3B_{2g}$	-294.543833	-295.624337	-295.634544	-295.580493
	$2\,^3B_{1g}$	-294.535020	-295.615004	-295.630473	-295.566969
	$2\,^3B_{3u}$	-294.511290	-295.587554	-295.609224	-295.530769
	$2\,^3B_{2u}$	-294.522654	-295.620923	-295.696220	-295.565203
Acetamide	$1\,^1A_g$	-208.071780	-208.894181	-208.894181	-208.842237
	$1\,^3A''$	-207.896576	-208.684965	-208.684965	-208.644276
	$1\,^3A'$	-207.871382	-208.658081	-208.669219	-208.618770
Formaldehyde	$1\,^1A_g$	-113.936026	-114.337900	-114.337900	-114.308177
	$1\,^3A_2$	-113.815087	-114.197684	-114.197684	-114.179743
	$1\,^3A_1$	-113.753766	-114.109498	-114.132363	-114.100917
Propanamide	$1\,^1A_g$	-247.119315	-248.128976	-248.128976	-248.066906
	$1\,^3A''$	-246.943445	-247.918603	-247.918603	-247.856208

Molecule	State	CASSCF	AC0	AC0D	AC
	$1\,^3A'$	-246.918558	-247.892110	-247.903176	-247.858372
Formamide	$1\,^1A_g$	-169.015993	-169.652919	-169.652919	-169.603346
	$1\,^3A''$	-168.842567	-169.446473	-169.446473	-169.398053
	$1\,^3A'$	-168.818838	-169.422048	-169.435099	-169.4010967
Acetone	$1\,^1A_g$	-192.050712	-192.825813	-192.825813	-192.792015
	$1\,^3A_2$	-191.940623	-192.659825	-192.659825	-192.640049
	$1\,^3A_1$	-191.879611	-192.588445	-192.596682	-192.569605
p-Benzoquinone	$1\,^1A_g$	-379.477673	-380.864233	-380.864233	-380.780829
	$1\,^3B_{1g}$	-379.374829	-380.758501	-380.759101	-380.681336
	$1\,^3A_u$	-379.374715	-380.758043	-380.758748	-380.681006
	$1\,^3B_{1u}$	-379.377088	-380.751655	-380.751655	-380.673442
	$1\,^3B_{3g}$	-379.343055	-380.720735	-380.735005	-380.642428

TABLE S3: Oscillator strengths in dependence on Dunning basis set: AVDZ, AVTZ, AVQZ and AV5Z. CCSDT results from respective bases, ref. [3]. FCI results for aug-cc-AVTZ basis, from ref. [4]

		$\mathbf{d}_\nu^{\mathrm{CAS}}$			$\mathbf{d}_\nu^{(0)}$		$\mathbf{d}_\nu^{(1)}$			
		CASSCF	AC0	AC0D	AC0	AC0D	AC0	AC0D	CCSD	FCI
					AVDZ					
Formamide	$2\,^1A'$	0.533	0.566	0.522	0.488	0.449	0.259	0.239	-	0.251
	$3\,^1A'$	0.218	0.232	0.214	0.308	0.284	0.147	0.135	-	-
Formaldehyde	$2\,^1A_1$	0.093	0.087	0.079	0.461	0.417	0.216	0.195	0.157	0.135
Ethene	$1\,^1B_{1u}$	0.403	0.405	0.395	0.403	0.392	0.351	0.342	0.365	0.346
					AVTZ					
Formamide	$2\,^1A'$	0.531	0.565	0.520	0.487	0.448	0.257	0.236	-	0.251

	$3\,^1A'$	0.204	0.215	0.193	0.312	0.280	0.092	0.082	-	-
Formaldehyde	$2\,^1A_1$	0.106	0.099	0.090	0.459	0.414	0.222	0.200	0.139	0.135
Ethene	$1\,^1B_{1u}$	0.400	0.404	0.393	0.401	0.391	0.346	0.337	0.362	0.346
					AVQZ					
Formamide	$2\,^1A'$	0.530	0.565	0.520	0.487	0.448	0.256	0.236	-	0.251
	$3\,^1A'$	0.218	0.232	0.214	0.308	0.283	0.077	0.071	-	-
Formaldehyde	$2\,^1A_1$	0.105	0.099	0.089	0.457	0.413	0.234	0.211	0.125	0.135
Ethene	$1\,^1B_{1u}$	0.398	0.403	0.392	0.401	0.390	0.343	0.334	0.360	0.346
					AV5Z					
Formamide	$2\,^1A'$	0.397	0.423	0.369	0.487	0.424	0.256	0.223	-	0.251
	$3\,^1A'$	0.106	0.112	0.101	0.307	0.276	0.056	0.050	-	-
Formaldehyde	$2\,^1A_1$	0.105	0.099	0.078	0.457	0.352	0.234	0.185	-	0.135
Ethene	$1\,^1B_{1u}$	0.396	0.402	0.391	0.400	0.389	0.340	0.331	-	0.346

TABLE S4: Singlet-triplet gaps. All values in eV. ^{a} CASPT2 results from Refs [5–19]. ^{b} CASPT2 results from Ref. [1]

Molecule	State	CASSCF	AC0	AC	CASPT2^a	CASPT2^b	CC2	CCSD	CC3	NEVPT2
Ethene	$1\,^3B_{1u}$	3.78	4.54	4.30	4.60	4.60	4.52	4.42	4.48	4.60
E-butadiene	$1\,^3B_u$	2.77	3.43	3.22	3.20	3.34	3.34	3.25	3.32	3.38
All-E-hexatriene	$2\,^3A_g$	2.66	2.81	2.77	2.55	2.71	2.78	2.62	2.69	2.73
All-E-octatetraene	$1\,^3B_u$	2.25	2.50	2.41	2.17	2.33	2.40	2.23	2.30	2.32
Cyclopropene	$1\,^3B_2$	3.78	4.64	4.61	4.18	4.35	4.44	4.30	4.34	4.56
Cyclopentadiene	$1\,^3B_2$	2.75	3.36	3.15	3.15	3.28	3.36	3.18	3.25	3.32
Norbornadiene	$1\,^3A_2$	3.07	4.03	3.70	3.42	3.75	3.76	3.67	3.72	3.79
Benzene	$1\,^3B_{1u}$	3.74	4.42	4.25	3.89	4.17	4.31	3.94	4.12	4.32
Naphtalene	$1\,^3B_{2u}$	2.93	3.38	3.23	3.10	3.20	3.27	2.99	3.11	3.26
Furan	$1\,^3B_2$	3.54	4.32	4.06	3.99	4.17	4.68	4.41	4.48	4.33

Molecule	State	CASSCF	AC0	AC	CASPT2[a]	CASPT2[b]	CC2	CCSD	CC3	NEVPT2
Pyrrole	$1\,^3B_2$	3.95	4.68	4.44	4.27	4.52	4.68	4.41	4.48	4.73
Imidazole	$1\,^3A'$	4.42	5.03	4.87	4.49	4.65	4.89	4.62	4.69	4.77
Pyridine	$1\,^3A_1$	3.81	4.59	4.31	4.05	4.27	4.46	4.07	4.25	4.47
s-Tetrazine	$1\,^3B_{3u}$	2.43	2.02	2.24	1.45	1.56	1.86	1.99	1.89	1.64
Formaldehyde	$1\,^3A_2$	3.32	3.80	3.59	3.48	3.58	3.57	3.52	3.55	3.75
Acetone	$1\,^3A_2$	4.17	4.30	4.30	3.90	4.08	4.08	4.03	4.05	4.10
p-Benzoquinone	$1\,^3B_{1g}$	2.80	2.88	2.70	2.17	2.63	2.47	2.71	2.51	2.82
Formamide	$1\,^3A''$	4.72	5.62	5.50	5.34	5.40	5.39	5.32	5.36	5.64
Acetamide	$1\,^3A''$	4.77	5.69	5.67	5.24	5.53	5.42	5.39	5.42	5.52
Propanamide	$1\,^3A''$	4.79	5.72	5.69	5.28	5.44	5.44	5.41	5.45	5.54

TABLE S5: Mean error (ME), mean unsigned error (MUE) and standard deviation (STD. DEV.) evaluated with respect to NEVPT2 and CC3 references for singlet-triplet gap for each molecule. All values in eV.

	CASSCF	AC0	AC	CASPT2[a]	CASPT2[b]	CC2	CCSD	CC3	NEVPT2
				Errors with respect to CC3					
ME	-0.41	0.23	0.11	-0.18	0.00	0.08	-0.05		0.11
MUE	0.49	0.24	0.21	0.19	0.07	0.09	0.08		0.15
STD. DEV.	0.39	0.14	0.32	0.14	0.12	0.08	0.08		0.14
				Errors with respect to NEVPT2					
ME	-0.52	0.12	0.01	-0.28	-0.10	-0.02	-0.16	-0.11	
MUE	0.59	0.13	0.20	0.28	0.10	0.11	0.20	0.15	
STD. DEV.	0.42	0.13	0.30	0.14	0.08	0.15	0.17	0.14	

Molecule	State	CASSCF	AC0	AC	CASPT2^a	CASPT2^b	CC2	CCSD	CC3	NEVPT2

[1] M. Schreiber, M. R. Silva-Junior, S. P. A. Sauer, and W. Thiel, J. Chem. Phys. **128**, 134110 (2008).

[2] I. Schapiro, K. Sivalingam, and F. Neese, J. Chem. Theor. Comp. **9**, 3567 (2013).

[3] A. Chrayteh, A. Blondel, P.-F. Loos, and D. Jacquemin, Journal of Chemical Theory and Computation **17**, 416 (2021), pMID: 33256412, https://doi.org/10.1021/acs.jctc.0c01111, URL `https://doi.org/10.1021/acs.jctc.0c01111`.

[4] M. Véril, A. Scemama, M. Caffarel, F. Lipparini, M. Boggio-Pasqua, D. Jacquemin, and P.-F. Loos, Wiley Interdisciplinary Reviews: Computational Molecular Science **11**, e1517 (2021).

[5] J. Finley, P.-Å. Malmqvist, B. O. Roos, and L. Serrano-Andrés, J. Phys. Chem. Lett. **288**, 299 (1998).

[6] L. Serrano-Andrés, M. Merchán, I. Nebot-Gil, R. Lindh, and B. O. Roos, J. Chem. Phys. **98**, 3151 (1993).

[7] L. Serrano-Andres, R. Lindh, B. O. Roos, and M. Merchan, J. Phys. Chem. **97**, 9360 (1993).

[8] R. González-Luque, M. Merchán, and B. Roos, Zeitschrift für Physik D Atoms, Molecules and Clusters **36**, 311 (1996).

[9] L. Serrano-Andres, M. Merchan, I. Nebot-Gil, B. O. Roos, and M. Fulscher, J. Am. Chem. Soc. **115**, 6184 (1993).

[10] B. O. Roos, M. Merchan, R. McDiarmid, and X. Xing, J. Am. Chem. Soc. **116**, 5927 (1994).

[11] J. Lorentzon, M. P. Fuelscher, and B. O. Roos, J. Am. Chem. Soc. **117**, 9265 (1995).

[12] M. Rubio, M. Merchán, E. Ortí, and B. O. Roos, Chem. Phys. **179**, 395 (1994).

[13] L. Serrano-Andrés, M. P. Fülscher, B. O. Roos, and M. Merchán, J. Phys. Chem. **100**, 6484 (1996).

[14] J. Lorentzon, M. Fülscher, and B. Roos, Theor. Chim. Acta **92**, 67 (1995).

[15] M. Rubio and B. O. Roos, Mol. Phys. **96**, 603 (1999).

[16] M. Merchán and B. O. Roos, Theor. Chim. Acta **92**, 227 (1995).

[17] M. Merchán, B. O. Roos, R. McDiarmid, and X. Xing, J. Chem. Phys. **104**, 1791 (1996).

[18] R. Pou-Amérigo, M. Merchán, and E. Ortı, J. Chem. Phys. **110**, 9536 (1999).

[19] L. Serrano-Andrés and M. P. Fülscher, J. Am. Chem. Soc. **118**, 12190 (1996).
